# Involvement of a velvet protein ClVelB in the regulation of vegetative differentiation, oxidative stress response, secondary metabolism, and virulence in *Curvularia lunata*

**DOI:** 10.1038/srep46054

**Published:** 2017-04-10

**Authors:** Jin-Xin Gao, Chuan-Jin Yu, Meng Wang, Jia-Nan Sun, Ya-Qian Li, Jie Chen

**Affiliations:** 1 School of Agriculture and Biology, Shanghai Jiaotong University, 800 Dongchuan Road, Shanghai 200240, P. R. China; 2 State Key Laboratory of Microbial Metabolism, Shanghai Jiaotong University, 800 Dongchuan Road, Shanghai 200240, P. R. China; 3 Ministry of Agriculture Key Laboratory of Urban Agriculture (South), Shanghai Jiao Tong University, 800 Dongchuan Road, Shanghai 200240, P. R. China

## Abstract

The ortholog of *Aspergillus nidulans* VelB, which is known as ClVelB, was studied to gain a broader insight into the functions of a velvet protein in *Curvularia lunata*. With the expected common and specific functions of ClVelB, the deletion of *clvelB* results in similar though not identical phenotypes. The pathogenicity assays revealed that ΔClVelB was impaired in colonizing the host tissue, which corresponds to the finding that ClVelB controls the production of conidia and the methyl 5-(hydroxymethyl) furan-2-carboxylate toxin in *C. lunata*. However, the deletion of *clvelB* led to the increase in aerial hyphae and melanin formation. In addition, ΔClVelB showed a decreased sensitivity to iprodione and fludioxonil fungicides and a decreased resistance to cell wall-damaging agents and osmotic stress and tolerance to H_2_O_2_. The ultrastructural analysis indicated that the cell wall of ΔClVelB became thinner, which agrees with the finding that the accumulated level of glycerol in ΔClVelB is lower than the wild-type. Furthermore, the interaction of ClVelB with ClVeA and ClVosA was identified in the present research through the yeast two-hybrid and bimolecular fluorescence complementation assays. Results indicate that ClVelB plays a vital role in the regulation of various cellular processes in *C. lunata*.

The *Curvularia* leaf spot (CLS) caused by the filamentous fungus *Curvularia lunata* (Wakker) Boedijn is one of the most widely distributed maize leaf diseases worldwide^1^. Aside from the large economic losses caused by *C. lunata*, the 5-(hydroxymethyl) furan-2-carboxylate (M5HF2C) toxin produced by the fungus in infected maize grains brings a severe threat to the health of humans and animals^2^. However, very little success has been achieved in developing effective control strategies for CLS. Therefore, a deep understanding of fungal development, M5HF2C biosynthesis, and the virulence of *C. lunata* could lead to the discovery of effective control strategies for this disease.

Previous studies have shown that the *clt-1* gene (accession: GQ292557) from the pathogen is closely associated with M5HF2C production^3^. Melanin is known to consolidate the mechanical penetration structures of phytopathogens, such as appressoria and infection pegs, that are required for effective penetration. Melanin has also been confirmed to belong to the virulence factor that can enforce the mechanical strength of infection into the epidermis of the host plant in numerous plant diseases. Moreover, several genes, including *brn1, brn2*, and *scd*, have also been cloned and evaluated based on their functions in the production of melanin^4^. In addition, *clt-1* and *brn1* may have some connection or a coordinated mediation mechanism in secondary metabolism. However, we do not know how both are connected^3^.

In addition to the virulence genes described above, various regulatory systems evidently control the regulation of the secondary metabolite biosynthesis in most fungi in response to the external environment^5^. The velvet family protein plays a key role in regulating secondary metabolism and the differentiation processes, such as fungal development and sporulation. It shares a common domain that is present in most parts of filamentous fungi. Different velvet protein members interact with each other in the nucleus^6^. As a key member of the velvet protein family, VelB has been researched in a few fungal species. In *Aspergillus nidulans*, the removal of *velB* leads to reduced secondary metabolites and sexual fruit body formation^6^. In *Fusarium fujikuroi*, FfVel2 (VelB ortholog) have similar functions in regulating fungal development and secondary metabolism^7^. Similar phenotypes of conidiation, melanin biosynthesis, hypersensitivity to oxidative stress, and virulence have been reported for the BcVelB (VelB ortholog) mutant^8^. This study aims to elucidate the functions of the VelB-ortholog ClVelB in *C. lunata*. In the current study, the deletion of *clvelB* showed a few distinct phenotypic characteristics compared with the VelB mutants in a few other fungi.

## Results

### Identification of the VelB ortholog in *C. lunata*

**Figure 1:**
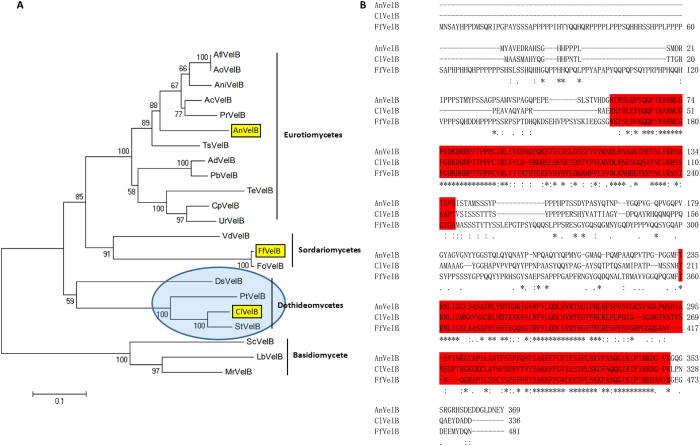
*C. lunata* VelB is an ortholog of A. nidulans VelB. (**A**) Phylogenetic analysis. VelB protein sequences were obtained from GenBank using *A. nidulans*. AnVelB as a query. AnVelB, *C. lunata* ClVelB, and *Fusarium fujikuroi* FfVelB are marked in yellow highlights. A blue oval shadow marks the single candidate ortholog. (**B**) ClVelB, AnVelB, and FfVelB were aligned using ClustalW. Conserved velvet superfamily domains are highlighted in red, asterisks mark identical residues, colons mark conserved residues, and periods indicate semi-conserved residues.

The ClVelB (accession number: KY435512) sequence was extracted from *C. lunata* genomic database (Dryad Digital Repository) using BlastP analyses with the sequence of *A. nidulans* VelB. The open reading frame of *clvelB* comprises 1,011 bp, does not contain introns, and encodes a 336-amino-acid protein. ClVelB falls in a group of dothideomycete VelB homologs, which is a sister to the eurotiomycete group including *A. nidulans* VelB, and the sordariomycete group including *F. fujikuroias* FfVel2 (Fig. 1A). The alignment of ClVelB with *A. nidulans* VelB (Fig. 1B) showed 90% positives and 51% identity (National Center for Biotechnology Information, BlastPAlign).

### Involvement of ClVelB in the regulation of hyphal growth, asexual development, and pigment formation in *C. lunata*

**Figure 2:**
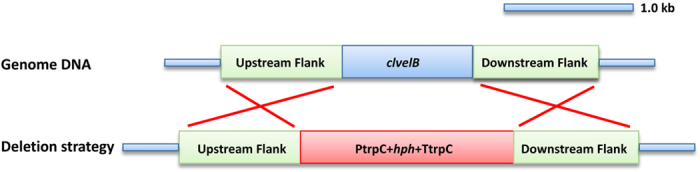
ClvelB deletion strategy used by homologous recombination. *clvelB* and hygromycin resistance (*hph*) genes are represented by blue and red boxes, respectively.

**Figure 3:**
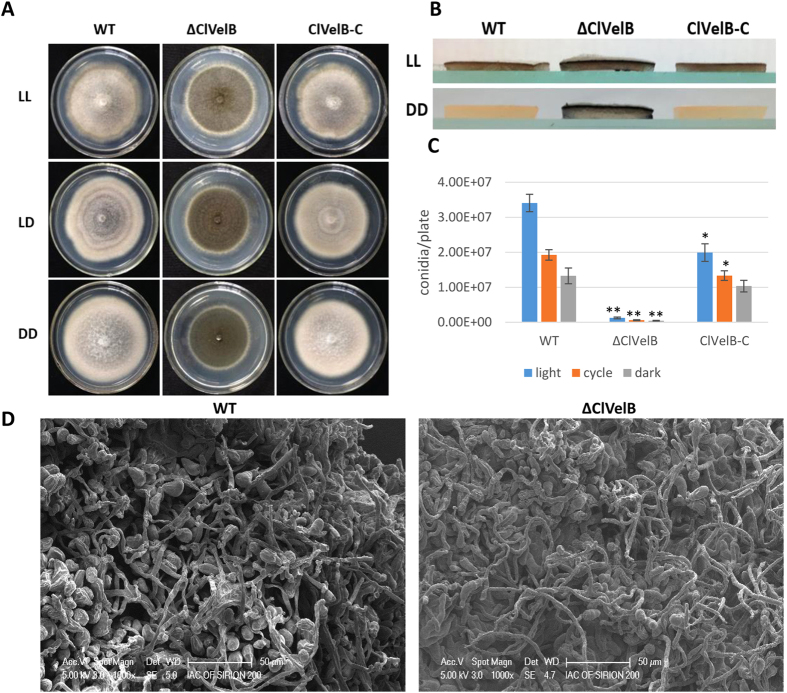
Effects of ClVelB on colony morphology and sporulation. (**A**) Cultures grown on CM plates under constant light (LL) or dark (DD), and 12 h light/dark cycle (LD) conditions for 7 days at 28 °C. Note that in LL, WT and ClVelB-C are white and flat, while ΔClVelB is pigmented and fluffy, which reflects aerial hyphal growth. Alternating banding rhythm in the middle plate suggests that the conidiation of WT is responsive to light. This banding rhythm is greatly reduced in ΔClVelB. (**B**) Side view of the plates of WT, ΔClVelB, and ClVelB-C grown in LL or DD on CM. Note the aerial hyphae on the plates of ΔClVelB, especially from LL. By contrast, the surface of the WT and ClVelB-C only shows a few aerial hyphae. (**C**) Quantification of conidia from cultures grown under LL, LD, and DD conditions. Error bars are the standard deviation. A single asterisk indicates the p-value < 0.05 while double asterisks indicate the p-value < 0.001 in the T-test analysis. Sporulation of ΔClVelB is repressed in all circumstances, while this was not observed for WT and ClVelB-C. (**D**) Hyphae structures of WT and ΔClVelB were examined through scanning electron microscopy (Sirion 200, FEI).

Target gene deletion strategy was employed by replacing *clvelB* with a hygromycin resistance (*hph*) cassette to investigate the biological functions of ClVelB in *C. lunata* (Fig. 2). The Southern hybridization pattern confirmed that homologous recombination occurs at the *clvelB* locus in ΔClVelB. Complementation of the deletion mutant (ClVelB-C) was accomplished by the reintroduction of wild-type (WT) *clvelB* into the genome of ΔClVelB. The radial growth rates of the mutants and WT on the complete medium (CM) under different light conditions (constant light [LL] or dark [DD], and 12 hours of light/dark photoperiod [LD]) were compared. ΔClVelB had a significantly slower mycelial growth rate than WT and the complemented strain ClVelB-C on the CM medium (Table 1). As the primary source of inoculum for host infections, conidia are formed during exposure to light. Time course experiments were performed to follow the onset of conidiation in the generated mutant under different illumination conditions. WT and ClVelB-C exhibited an obvious banding rhythm which reflected periods of conidiation under LD conditions, whereas that in ΔClVelB was greatly reduced (Fig. 3A). The conidiation of WT was the most in the LL condition, the least in the DD condition, and a moderate number in the LD condition (Fig. 3C). However, the conidiation of ΔClVelB sharply declined, and the differences in conidiation in the preceding three conditions were not as obvious as in WT and ClVelB-C (Fig. 3C).

WT and ClVelB-C sparingly developed aerial hyphae accompanied by high numbers of conidia in the LL condition. Although the hyphae of ΔClVelB were not evidently different from those of the WT (Fig. 3D), ΔClVelB exhibited “fluffy” colonies that are characterized by a cotton-like appearance (Fig. 3B) and produced fewer conidia (Fig. 3C). Overall, these results indicate that ClVelB controls the balance between aerial conidiation and hyphal growth, that is, it represses aerial hyphae growth and promotes conidiation in response to the light condition.

### ClVelB regulates the melanization of mycelia

**Figure 4:**
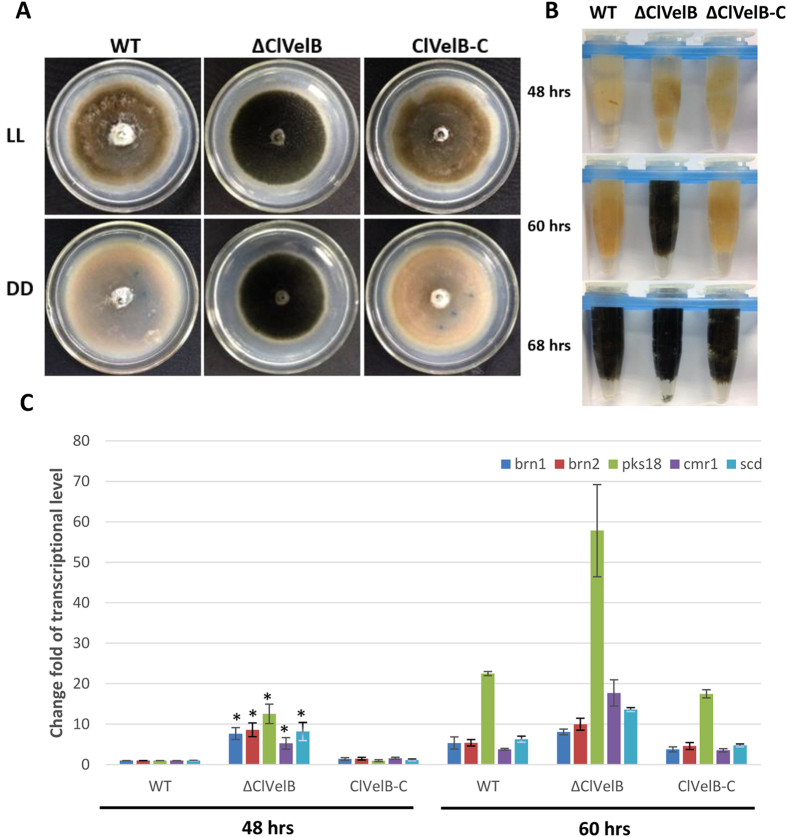
ClVelB negatively regulates the mycelial melanization of *C. lunata*. (**A**) Bottom of the CM plates of WT strain (CX-3), *clvelB* deletion mutant (ΔClVelB), and complemented strain (ClVelB*-*C) grown in constant light (LL) or dark (DD) for 7 days. Photos were taken after removing conidia. Note the heavy melanization of mycelia of ΔClVelB in both LL and DD compared to WT. (**B**) The mycelial pellet of WT, ΔClVelB, and ClVelB*-*C at different indicated time points. ΔClVelB is melanized by 60 h, which is ahead of WT and ClVelB*-*C. Pigmentation starts by 68 h in WT and ClVelB*-*C. (**C**) qRT-PCR analyses of *pks18, cmr1, brn1, brn2,* and *scd*. Expression was tested at 48 and 60 h. The expression level compared with the WT at 48 h is shown. Error bars are the standard deviation. A single asterisk indicates the p-value < 0.05 in a T-test analysis.

**Figure 5:**
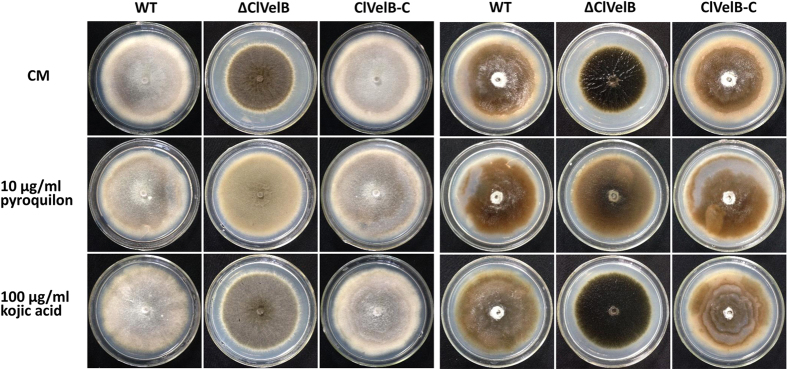
DHN type melanin produced by *C. lunata*. (**A**) 10 μg/ml pyroquilon or 100 μg/ml kojic acid were added into the CM to confirm that the conidial and mycelial melanin of *C. lunata* is not the tyrosine-derived but DHN type. (**B**) Culture plates after removing conidia. The mycelial color had gone from black to light brown for all strains detected on the pyroquilon plates, but no change on the kojic acid medium.

Similar to *Botrytis cinerea* BcVelB, the deletion of ClVelB leads to an increase in mycelial pigmentation^8^, and insufficient ClVelB leads to the increased melanization of mycelia, which is grown both on a solid (Fig. 4A) and in a liquid CM medium (Fig. 4B), indicating that ClVelB negatively regulates the mycelial pigmentation in *C. lunata*. Hyphal pigmentation develops faster in ΔClVelB than in WT (Fig. 4B). By 68 h, all strains were darkly pigmented. We detected the expression of the PKS gene (*pks18*), the transcription factor gene (*cmr1*), and three synthase genes (*brn1, brn2,* and *scd*) related to the synthesis of DHN melanin in the WT and mutant to further confirm this observation (Fig. 4C)^4^,^9^. qRT-PCR analyses showed that the expression levels of *pks18* in ΔClVelB were enhanced compared to those in WT. By 48 h, the expression of *pks18* in ΔClVelB has a 12.53-fold increase, which peaked at 60 h (57.82-fold). At 48 h, the expression of *cmr1* has a 5.25-fold increase in ΔClVelB compared to that in WT. *brn1, brn2,* and *scd* also showed high expression levels in ΔClVelB compared to those in WT at both 48 and 60 h. For all the genes, the reintroduction of *clvelB* restored the WT expression levels. Overall, we conclude that ClVelB plays a negative regulation role in the synthesis of melanin. The pyroquilon and kojic acid inhibitors were used to study the influence on melanization and support our previous studies that the conidial and mycelial melanin of *C. lunata* is not the tyrosine-derived but DHN type^10^. While the colors of all the cultures (WT, ΔClVelB, and ClVelB-C) grown on kojic acid remained the same, those grown on pyroquilon were changing from black to light brown (Fig. 5), bolstering previous research on melanization in *C. lunata*.

### ClVelB is required to cope with oxidative stress

**Figure 6:**
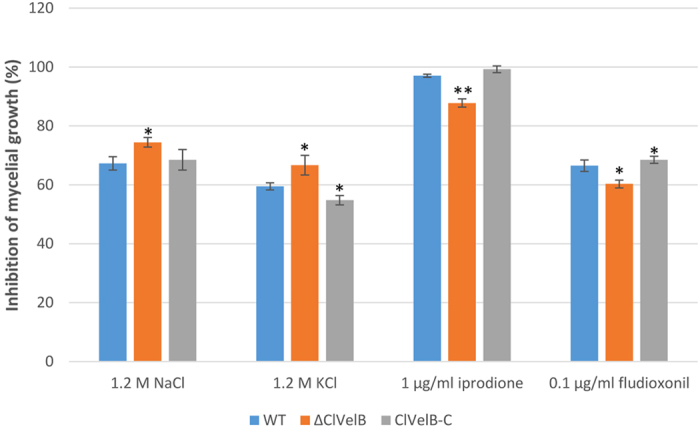
Sensitivity of the WT strain (CX-3), *clvelB* deletion mutant (ΔClVelB), and complemented strain (ClVelB-C) to the osmotic stresses and fungicides. 1.2 M NaCl or KCl were added into the CM to study the osmotic stresses. Iprodione or fludioxonil was added into the CM at a final concentration of 1 μg/ml or 0.1 μg/ml, respectively, to test the tolerance of fungicides. Error bars are the standard deviation. (**A**) Single asterisk indicates the p-value < 0.05 while double asterisks indicate the p-value < 0.001 in the T-test analysis.

**Figure 7:**
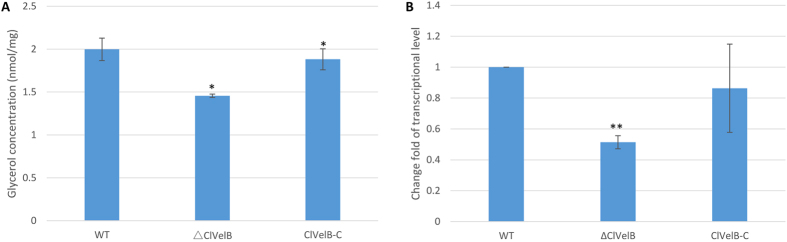
ClVelB regulates the glycerol accumulation. (**A**) Determination of glycerol biosynthesis in WT strain (CX-3), *clvelB* deletion mutant (ΔClVelB), and complemented strain (ClVelB-C). After the mycelia of WT, ΔClVelB and ClVelB-C were treated with 1.2 M NaCl for 2 h, the intracellular glycerol concentrations (nmol/mg dried mycelia) were tested. Untreated mycelia were used as the controls. The bars indicate the standard errors of the three repeated trails. (**B**) qRT-PCR analysis of *gpd1*, which is responsible for glycerol biosynthesis. A single asterisk indicates the p-value < 0.05 while double asterisks indicate the p-value < 0.001 in the T-test analysis.

**Figure 8:**
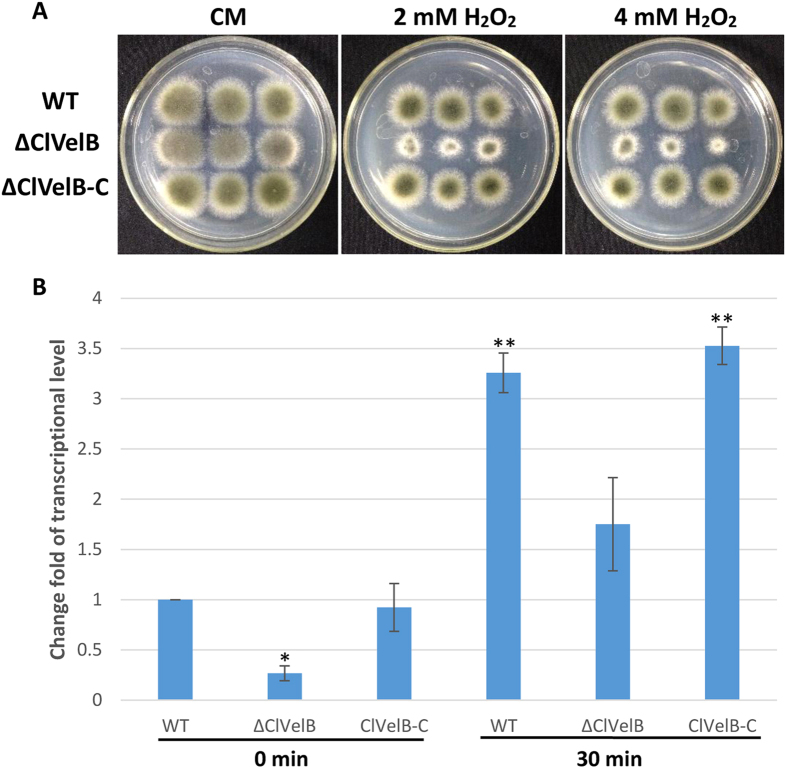
Sensitivity of the WT strain (CX-3), clvelB deletion mutant (ΔClVelB), and complemented strain (ClVelB-C) to oxidative stress. (**A**) 4, 2, and 1 μl conidial suspensions prepared from WT, ΔClVelB, and ClVelB-C were dripped on a CM plate with the with the indicated concentrations of H_2_O_2_. ΔClVelB is more sensitive to H_2_O_2_ than WT and ClVelB-C. (**B**) qRT-PCR analysis of the catalase-encoding gene *cat3*. Error bars are the standard deviation. A single asterisk indicates the p-value < 0.05 while double asterisks indicate the p-value < 0.001 in a T-test analysis. The expression levels of *cat3* were reduced in ΔClVelB (3.7-fold at time 0 and 3.3-fold 30 min after H_2_O_2_ addition).

The growth rates of the mutants were quantified on media supplemented with stressors that induce osmotic stress (1.2 M NaCl, 1.2 M KCl), fungicides (10 μg/mL iprodione, 0.1 μg/mL fludioxonil), and oxidative stress (2.0 or 4.0 mM H_2_O_2_) to assess whether ClVelB is also essential to cope with various kinds of stresses. Under osmotic stress conditions, all mutants showed comparable growth rates. ΔClVelB showed a slightly decreased resistance to osmotic stresses cultured in 1.2 M NaCl or 1.2 M KCl medium and a slight decreased sensitivity to the dicarboximide fungicide iprodione and phenylpyrrole fungicide fludioxonil (Fig. 6). The intercellular glycerol of fungus plays a significant role in responding to osmotic stress^11^. As shown in Fig. 7, ΔClVelB exhibited a low basal level of glycerol accumulation and the expression of the *gpd1* gene that is responsible for glycerol synthesis showed a similar trend, which partially explains why ΔClVelB exhibited decreased resistance to osmotic stresses. ΔClVelB showed high sensitivity to H_2_O_2_ compared to the WT strain, reintroduction of WT *clvelB* gene into the mutant restored the tolerance of WT to oxidative stress (Fig. 8A).

The expression of the catalase gene *cat3* that related to oxidative stress responses, exhibited an obvious difference between the WT and the *clvelB* mutant (Fig. 8B). In ΔClVelB, *cat3* decreased by approximately 3.7-fold before adding H_2_O_2_ and approximately 3.3-fold after the addition of H_2_O_2_ compared to the level in the WT strain. Collectively, the data indicate that ClVelB regulates oxidative stress responses by controlling the expression of *cat3* gene. The mechanism should be researched further.

### ClVelB regulates cell wall integrity

**Figure 9:**
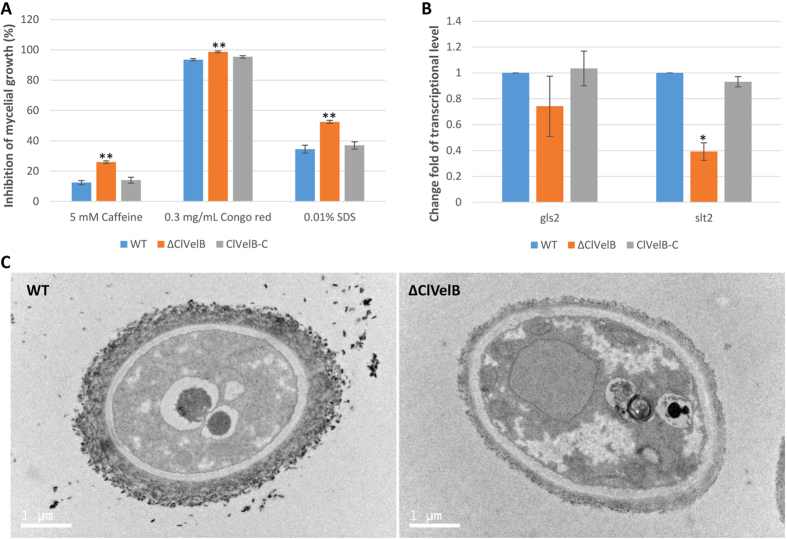
ClVelB regulates cell wall integrity. (**A**) Sensitivity of the WT strain (CX-3), *clvelB* deletion mutant (ΔClVelB), and complemented strain (ClVelB-C) to the cell wall damaging agents. The detection was made on a CM plate added with the corresponding cell wall damaging agent. (**B**) Expression changes of *gls2* and *slt2* in each strain. The relative expression levels of *gls2* and *slt2* in ΔClVelB are the relative cDNA amounts of the same gene in the WT strain. Line bars indicate the standard errors from the three trial replicates. A single asterisk indicates a p-value < 0.05 while double asterisks indicate a p-value < 0.001 in the T-test analysis. (**C**) Ultrastructural analyses of the cell of the *clvelB* deletion mutant. Cells of the WT and ΔClVelB were observed with a transmission electronic microscope (Tecnai G2 Spirit Biotwin, FEI). Mycelia were harvested and fixed in glutaraldehyde for 12 h at 4 °C.

The deletion of *clvelB* led to a decrease in resistance to osmotic stresses, which indicates that ClVelB might regulate the integrity of the cell wall and/or the cell member. To prove this hypothesis, we tested the sensitivity of ΔClVelB to cell wall damaging agents, namely, Congo red and Caffeine and to cell member damaging agent SDS. The results indicate that ΔClVelB displayed a decreased resistance to these compounds to some extent (Fig. 9A). Studies have shown that Congo red could disturb the fungal cell wall by binding to cellulose and chitin^12^. Thus, we tested the expressions of the 1,3-beta-glucan synthase gene *gls2* and MAPK gene *slt2*, which are homologous to the core element genes of *Saccharomyces cerevisiae* cell wall integrity (CWI) pathway, in the *clvelb* deletion mutant. The expression levels of *gls2* and *slt2* in ΔClVelB were lower than those in WT (Fig. 9B), which agrees with the phenotype that ΔClVelB showed decreased resistance to Congo red. More interestingly, we found that the deletion of *clvelB* led to the decrease of fungal cell width compared with WT (Fig. 9C). These results demonstrate that ClVelB might be related to the regulation of the CWI pathway in *C. lunata*.

### Effects of ClVelB on the hyphal hydrophobicity

**Figure 10:**
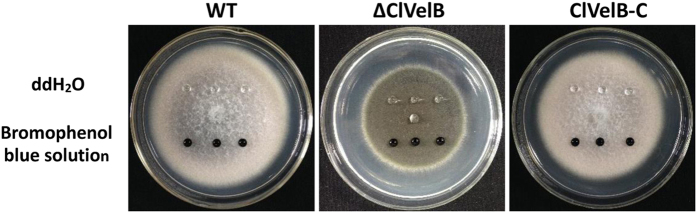
Effects of the clvelB deletion on hyphal hydrophobicity. 20 μl of ddH_2_O or 2.5% bromophenol blue solution was dropped on the colony surfaces of the WT strain (CX-3), *clvelB* deletion mutant (ΔClVelB), and complemented strain (ClVelB-C), and photographed 10 min later. The droplet did not disperse on the colony of ΔClVelB, WT, and ClVelB-C.

In numerous fungal species, the cell surface of aerial hyphae shows a distinct hydrophobic feature^13^. The deletion of *fgvelB* leads to loss of function to maintain the hydrophobicity of the hyphal surface in *Fusarium graminearum*^14^. To confirm if *clvelB* has the same function in *C. lunata*, 20 μl drops of 2.5% bromophenol blue solution or ddH_2_O were added to each strain surface. Both the 2.5% bromophenol blue solution and the ddH_2_O maintained spherical droplets on the surface of the ΔClVelB colony without being absorbed or extended for more than 30 min, thereby demonstrating the strong hydrophobicity of the ΔClVelB hyphae, which is similar to those of WT, and the complemented strain (Fig. 10). These results indicate that ClVelB did not contribute in regulating the hyphal hydrophobicity of *C. lunata*.

### ClVelB regulates M5HF2C toxin biosynthesis

**Figure 11:**
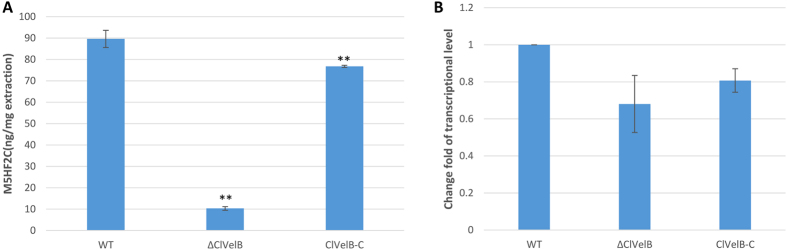
ClVelB regulates the biosynthesis of M5HF2C toxin. (**A**) Amount of M5HF2C (per mg extraction) produced by WT strain (CX-3), *clvelB* deletion mutant (ΔClVelB), and complemented strain (ClVelB-C) that were cultured in Fries 3 medium after 30 days. (**B**) qRT-PCR analysis of the M5HF2C biosynthesis related gene *clt-1* in WT, ΔClVelB, and ClVelB-C. Error bars are the standard deviation. A single asterisk indicates the p-value < 0.05 while double asterisks indicate the p-value < 0.001 in a T-test analysis.

Reports indicate that VelB regulates the synthesis of secondary metabolites in numerous fungi^15^. Therefore, detecting the M5HF2C toxin production in ΔClVelB is necessary. After culturing in Fries 3 medium for 30 days, the amount of M5HF2C produced by ΔClVelB was 79.3% lower than that produced by WT (Fig. 11A). The expression of the M5HF2C biosynthesis related gene *clt-1* was analyzed by qRT-PCR to further confirm that ClVelB acts as a positive regulator of M5HF2C toxin production. The expression level of *clt-1* in ΔClVelB decreased by 31.9% compared to that in WT, which was consistent with the profiles of M5HF2C production (Fig. 11B). The experiment results indicate that ClVelB played a major role in the regulation of M5HF2C biosynthesis in *C. lunata*.

### ClVelB is essential for virulence in *C. lunata*

**Figure 12:**
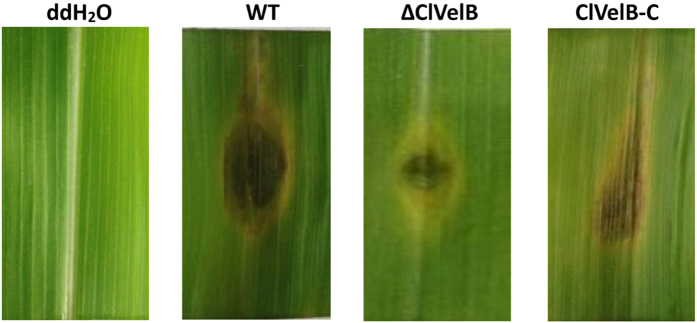
Virulence of the WT (CX-3), clvelB deletion mutant (ΔClVelB), and complemented strain (ClVelB-C) on maize leaves. *clvelB* deletion mutants are impaired in the colonization of maize leaves. Detached leaves of HUANGZAO-4 were inoculated with conidial suspensions and incubated on two layers of filter papers moisturized with 10 mM 6-Benzyladenine (6-BA) in Petri dishes at 28 °C for 96 h.

Mycotoxin M5HF2C has been described as one of most important virulence factors in *C. lunata*^2^. We further assayed the infective ability of ΔClVelB on maize leaves because the deletion of *clvelB* compromised the ability of *C. lunata* to produce M5HF2C. The penetration and establishment of primary lesions by ΔClVelB were similar to those by WT. However, the infection proceeded differentially. The capability of ΔClVelB to colonize the surrounding host tissue was impaired (Fig. 12). In any case, the lesion sizes on maize leaves inoculated with ΔClVelB decreased significantly compared to those inoculated with WT, indicating that ClVelB was essential to the complete virulence in *C. lunata*.

### Interaction of ClVelB with ClVeA and ClVosA in *C. lunata*

**Figure 13:**
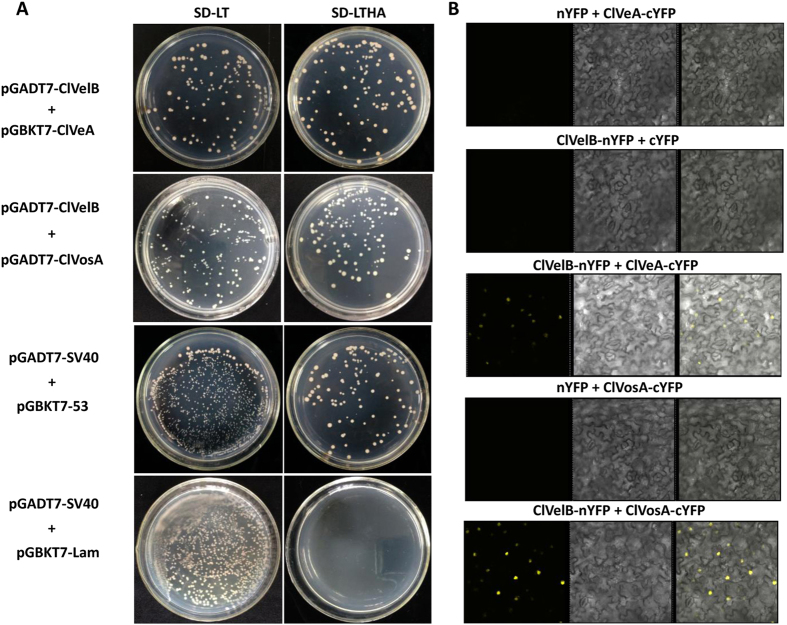
Interaction of ClVelB with ClVeA and ClVosA in *C. lunata*. (**A**) ClVelB interacts with ClVeA and ClVosA in a Y2H approach. For Y2H analysis, the *clvelb* cDNA was fused to the GAL4 activation domain, and the cDNAs of *clveA* and *clvosA* were respectively fused to the GAL4 binding domain. The cell suspensions of the *Saccharomyces cerevisiae* strain (AH109) containing the indicated vectors were dropped onto the selected media. SD-LT: synthetically defined (SD) medium without leucine and tryptophan was used to demonstrate the presence of both vectors. SD-LTHA: SD without leucine, tryptophan, histidine, and adenine supplemented with 11 mM 3-amino-1,2,4-triazole (SD_LWH+3-AT) was used to detect HIS3 reporter gene activity (displayed by colony growth). Plasmid pairs of pGADT7-SV40/pGBKT7-53 and pGADT7-SV40/pGBKT7-Lam served as the positive and negative controls, respectively. (**B**) Interaction analysis of ClVelB with ClVeA and ClVosA versions by BiFC assay in infiltrated *Nicotiana benthamiana* leaves. Co-expression of ClVelB with ClVeA and ClVosA versions fused to splitYFP. n/cYFP: n- or c-terminal part of splitYFP.

In *A. nidulans*, the positive control of secondary metabolism is accomplished through the physical interaction of VelB with another velvet-like protein VeA in the nucleus^6^, and VelB-VosA heterodimer has additional functions in trehalose biogenesis and spore viability^16^. A direct yeast two-hybrid (Y2H) method was used to ascertain the analogous protein-protein interactions of the *C. lunata* orthologs (ClVelB [336 aa], ClVeA [598 aa, accession number: KY435511], and ClVosA [302 aa, accession number: KY435513]). The full-length ClVelB protein was fused to the GAL4 activation domain, and the full-length proteins of ClVeA and ClVosA were respectively fused to the GAL4 binding domain. Then, yeast cells expressing different combinations were tested for ADE2 and HIS3 reporter gene activities. This experiment showed that ClVelB interacts with ClVeA and ClVosA (Fig. 13A). Bimolecular fluorescence complementation (BiFC) experiments with splitYFP-constructs were conducted to control the false positive fluorescence signal due to simple and close co-localization more stringently and further confirm the dimerization of ClVelB with ClVeA and ClVosA. The BiFC analysis suggests that ClVelB can interact with ClVeA and ClVosA as homodimers in the cellular nuclei of tobacco (Fig. 13B).

## Discussion

VelB has been reported to be a filamentous fungi-specific regulator that plays multifaceted roles in various biological processes, including fungal development, colonial morphology, and secondary metabolism. However, certain changes in the preceding roles have been found in different fungi. For example, the deletion of *Ffvel2* led to decreased conidiation and hyphal growth in *F. fujikuroi*^7^. In this research, the *clvelB* deletion mutant also exhibited reduced growth rate (Table 1) and conidiation (Fig. 3C) but increased aerial hyphae formation (Fig. 3B). In *F. graminearum*, the disruption of *velB* caused the hydrophobicity change of the cell surface^14^. Instead, we found that the *clvelB* deletion mutant exhibited no effect on hydrophobicity (Fig. 10). A recent study of *A. nidulans* indicated that the conidia of the *velB* mutant showed decreased resistance to numerous H_2_O_2_ and UV stresses, which resulted in a low-level accumulation of trehalose in the mutant^16^. In the current study, we also found that the deletion of *clvelB* led to the slightly decreased resistance to a few stress agents, including NaCl and KCl (Fig. 6), which may be attributed to a lower basal accumulation of glycerol in ΔClVelB compared with that in WT (Fig. 7). The reduced tolerance to stress agents in ΔClVelB indicated a variation in the cell membrane or cell wall composition. Therefore, we tested the sensitivity of ΔClVelB to the cell member damaging agent SDS and the cell wall damaging agents Caffeine and Congo red. In line with the expectations, ΔClVelB showed a decreased resistance to these compounds (Fig. 9A), which is in agreement with the expressions of the 1,3-beta-glucan synthase gene *gls2* and MAPK gene *slt2* in the *clvelB* deletion mutant (Fig. 9B). Moreover, the cell wall of ΔClVelB became thinner (Fig. 9C). These results demonstrate that ClVelB may regulate the cell wall composition and integrity in *C. lunata*, indicating that VelB can bind to the promoter region of the β-glucan synthase gene *fksA* to regulate the cell wall synthesis in *A. nidulans*^17^.

When tested for pathogenicity, ΔClVelB produced smaller lesions than WT or the complemented strain (Fig. 12). With regard to virulence and basic metabolism of fungal cells, we also found that the disruption of *clvelB* affects the redox status, the *clvelB* deletion mutant is more sensitive to H_2_O_2,_ and the growth defects became more evident (Fig. 8A). Managing ROS is a determinant of fungal success in infecting host and in the basic cellular of fungal cells. In accordance with the more pronounced effect of H_2_O_2_ on the radial growth rate of the *clvelB* mutant compared to that of the WT, a significant reduction in the expression level of *cat3* was observed (Fig. 8B). Reactive oxygen species (ROS) plays a major role in pathogen-host interactions^18^. Under a pathogen attack, plants use the oxidative burst as an initial defense reaction. The fungus shows resistance against oxidative burst while infecting the host plant because *C. lunata* has effective ROS-detoxification systems, such as peroxidases and catalases^19^. Thus, the increased sensitivity of the *clvelB* mutant to oxidative stress might be partially related to the reduced virulence of the mutant on the host plant.

VelB proteins have been reported to regulate secondary metabolism in some fungi. In *A. nidulans*, the *velB* deletion mutant showed decreased sterigmatocystin (SM) production and synthesized a brownish pigment^6^. In *F. graminearum*, the *FgVelB* mutant produced a yellow pigment and a dramatically low level of DON^14^. In the current study, we observed that ΔClVelB produced a significantly high level of melanin (Fig. 4A). Furthermore, the expressions of five DHN melanin biosynthesis genes were significantly up-regulated in ΔClVelB (Fig. 4C). These results indicate that VelB repressed melanin expression as previously described in *Cochliobolus heterostrophus*^20^. In contrast, the *clvelB* mutant produced a lower expression of M5HF2C toxin (Fig. 11A), which has been identified as one of the most important virulence factors of *C. lunata*^21^. ClVelB is essential for virulence to facilitate the colonization of the plant tissue. Notably, the penetration via germ tubes or infection cushions remains unaffected in the deletion mutant. Thus, no difference between the primary infections of ΔClVelB and WT exists. However, the lesions of ΔClVelB did not spread, suggesting that the mutant cannot kill the ambient host cells of the infection site. Predicting the reason for this result is difficult, and several factors are probably responsible. Hence, mycotoxin production and conidiation may contribute to virulence.

The velvet proteins of VeA, VelB, and VosA are fungi-specific transcription factors, which contain the velvet domain^22^. In numerous filamentous fungi, these proteins form different complexes that play distinct roles. Among them, VelB forms a heterodimer with VeA, which is required for secondary metabolites production and fungal deveploment^6^. In *A. nidulans*, the disruption of either *velB* or *veA* results in defects in the SM production and sexual fruiting body formation^22^. In the same way, FgVelB and FgVeA have similar roles in regulating fungal development, glycerol accumulation, DON synthesis, and pathogenicity^14^, which indicates that VelB cooperates with VeA to regulate fungal development and secondary metabolism. In *A. nidulans*, VelB contains neither a typical nuclear export signal (NES) nor a nuclear localization signal (NLS). Instead, the *A. nidulans* VeA protein includes a NES and a bipartite NLS in the N-terminal part. VeA is necessary for the efficient nuclear import of VelB. Earlier studies on *A. nidulans* have shown that the positive control of secondary metabolism can be achieved via the physical interaction of VelB with VeA in the nucleus^6^. VelB has additional functions in trehalose biogenesis and spore viability, which requires the VelB–VosA heterodimeric protein complex formation^23^. In *A. nidulans*, the VelB–VosA complex represses β-glucan synthesis by directly binding to the promoter regions of the cell wall biosynthetic genes in conidia and ascospores, thereby activating the formation of spore wall during sporogenesis^17^. The Y2H and BifC approaches confirmed that the *C. lunata* orthologs ClVelB interacted with ClVeA and ClVosA, and likely formed the complexes of ClVelB–ClVeA and ClVelB–ClVosA analogous to the situation found in other filamentous fungi^23^. Given that these complexes regulate numerous processes in fungal biology, we suspected that ClVelB may regulate the biosynthesis of M5HF2C toxin and DHN melanin in combination with ClVeA and interact with ClVosA to control the sporulation in *C. lunata*. Studying the roles of the ClVelB–ClVeA and ClVelB–ClVosA complexes in the different functions in *C. lunata* is interesting because ClVelB interacts with ClVeA and ClVosA in the Y2H and BiFC tests (Fig. 13). In conclusion, this study would help us understand the biological roles of *C. lunata* and may provide target sites for designing a new agent to control *C. lunata* and a few similar fungi.

## Methods

### Fungal strains, plant materials, and culture conditions

*C. lunata* WT strain CX-3, whose genome sequence is available (Dryad Digital Repository)^24^, was used as a progenitor for the transformation experiment in this study. The *clvelB* gene deletion strain was generated in the CX-3 genomic background. ClVelB-C was strain complemented with the WT *clvelB* gene. Unless mentioned otherwise, all strains were cultivated in Petri dishes containing solid synthetic CM medium. Cultures were incubated at 28 °C under constant light (LL) or dark (DD), and 12 h light/dark cycle (LD) conditions for conidiation. *Zea mays* cultivars (HUANGZAO-4) and *Nicotiana benthamiana* were grown under 16 h of light/8 h of darkness at 24 °C.

### Identification of VelB orthologs in *C. lunata*

The *A. nidulans* VelB (accession number: ABQ17967) and *F. fujikuroi* FfVel2 (accession number: FN675836) were used to query the *C. lunata* genome database for orthologs. Fungal genomic DNA and total RNA were prepared as previously described to verify the existence and sizes of introns in *clvelB*^25^. DNA and cDNA amplification were performed using the primer pair VelB-FL-F and VelB-FL-R, respectively (Table S1). Phylogenetic tree was built using the MEGA 5.0 and alignment created using ClustalW.

### *clvelB* gene deletion and complementation

We inserted two flanking sequences of *clvelB* into the two sides of the *hph* gene in pC1300 kh vector to construct the deletion vector 1300 kh-ClVelB-D (Fig. 2)^26^. *clvelB* was deleted using the ATMT method^3^. Hygromycin was added to the medium to a final concentration of 200 μg/ml for selecting transformants, and putative *clvelB* deletion mutants were verified by the PCR and Southern hybridization tests.

The pC1300N vector contains the G418 resistance cassette comprising the G418 resistance gene under the control of its promoter and the TrpC terminator from *A. nidulans* was used for gene complementation. The full-length sequence of *clvelB* under the control of the promoter and the TrpC terminator were inserted into the HindIII-XbaI sites of pC1300N to create plasmid 1300N-ClVelB-C and to construct ClVelB complementary mutants^26^. The final plasmid carrying both the WT *clvelB* and the G418 resistance cassette, as well as the TrpC terminator, was used to transform the *clvelB* deletion mutant and subsequently create a *clvelB*-restoring strain using the ATMT method as described above except for the use of the G418 selection agent. The integration at the target sites and the complementation of the *clvelB* mutant were confirmed through the PCR and Southern hybridization analysis. The sequences of primers for gene disruption, complementation and PCR confirmation are shown in Table S1.

### Analysis of mycelial development and conidiation

Mycelial development was observed under different conditions on a CM plate added with the corresponding agents that were suggested in the figure legends. Mycelial development was tested according to the description of the procedure^14^. The conidia that formed on the CM were harvested from the cultures of each strain with 5 ml of sterile ddH_2_O and were immediately counted with a hemocytometer. Each experiment was independently treated with three replications.

### Microscopic observation of conidial and hyphal morphology

The conidial and hyphal morphologies of each strain were examined using the electron microscope Tecnai G2 Spirit Biotwin (FEI, USA) and Hitachi Sirion 200 scanning electron microscope (FEI, USA), respectively. The samples were prepared according to the description of the methods^14^.

### Detection of intracellular glycerol content

Each strain was cultured in a liquid CM medium at 180 rpm for 72 h at 28 °C. After dealing with 1.2 M NaCl for 2 h, mycelia were collected and ground in liquid nitrogen. Mycelial powders (100 mg) were harvested to test the glycerol content using the glycerol assay kit (Chaoyan, Shanghai, China) according to the instructions of the manufacturer. Each experiment was independently replicated three times.

### Oxidative stress sensitivity tests

Tests of sensitivity to H_2_O_2_ and gene expression analyses were conducted as described^20^,^27^.

### Pigmentation

The pigmentation of hyphae on a solid medium and melanin types were tested according to the description of the procedure^20^,^28^,^29^. WT strain (CX-3), *clvelB* deletion mutant (ΔClVelB), and its complemented strain (ClVelB-C) were cultured in a liquid CM and transferred to 1.5 ml Eppendorf tubes at 48, 60, and 68 h to test the pigmentation of hyphae. The samples at 48 and 60 h were used for qRT-PCR analyses of *cmr1, pks18, brn1, brn2,* and *scd*. The genes were expressed as fold change compared with that of WT at 48 h.

### Analysis of M5HF2C toxin production and expression level of *clt-1*

The mutants were cultured in Fries 3 medium for 30 days to determine whether they retained the ability to produce the virulence-related toxin M5HF2C. HPLC-MS analysis of the extract from *C. lunata* cultures was performed on an Agilent 1100 high-pressure liquid chromatography station to determine the amount of M5HF2C using a previously described protocol^2^. The mycelia of WT, ΔClVelB, and ClVelB-C were inoculated into the liquid CM medium and cultured at 180 rpm for 72 h at 28 °C to determine the expression levels of *clt-1*. The total RNA was extracted and the expressive level of *clt-1* was determined using qRT-PCR assays^30^. Each experiment was independently replicated three times.

### Virulence assays

For infection assays, the fourth leaves of the susceptible maize HUANGZAO-4 seedlings at the seven-leaf stage were inoculated with 10 μl droplets of conidial suspensions (1.0 × 10^6^ conidia/ml). These inoculated leaves were incubated on two layers of Whatman 3MM filter papers moisturized with 10 mM of 6-benzyladenine (6-BA) in Petri dishes at 28 °C for 96 h. This test was independently replicated three times.

### Y2H assay

The full-length cDNA sequences of *clvelB, clveA*, and *clvosA* were amplified to verify the probable interaction of ClVelB with ClVeA and ClVosA using Y2H assay. The *clvelb* cDNA was inserted into the *EcoRI-BamHI* sites of the pGADT7 vector containing the yeast GAL4 activation domain, and the cDNAs of *clveA* and *clvosA* were respectively inserted into the *EcoRI-BamHI* sites of the pGBKT7 vector containing the GAL4 binding domain (Clontech, Mountain View, CA, USA). The plasmid pairs of pGADT7-ClVelB/pGBKT7-ClVeA and pGADT7-ClVelB/pGBKT7-ClVosA were co-transformed into the *S. cerevisiae* reporter strain AH109 using the LiAc/SS-DNA/PEG transformation method^31^. The plasmid pairs of pGADT7-SV40/pGBKT7-53 and pGADT7-SV40/pGBKT7-Lam served as the positive and negative controls, respectively. The experiment was independently replicated three times.

### BiFC assay

The *clvelB, clveA,* and *clvosA* cDNA sequences were cloned via the pDONR/Zeo vector and the Gateway cloning system (Life Technologies, CA, USA) into the corresponding splitYFP binary vectors (pEarleyGate202-YN or pEarleyGate201-YC). Agrobacteria EHA105 were transformed with the final constructs for the subsequent infiltration of *N. benthamiana* leaves. For transient expression experiments with (split) fluorescence protein fusion constructs, agrobacteria containing a construct with *clvelB* and agrobacteria containing *clveA* or *clvosA* were co-infiltrated. The co-infiltration experiments were performed in all appropriate combinations and repeated at least three times with similar results. The fluorescence signal was analyzed 48 h after infiltration by confocal laser scanning microscopy (Zeiss LSM 700, Zeiss, Germany).

## Additional Information

**Accession codes**: **Eurotiomycetes**: *Aspergillus clavatus* AcVelB: XP_001269945; *Ajellomyces dermatitidis* AdVelB: XP_002625924; *Aspergillus flavus* AfVelB: XP_002373064; *Aspergillus nidulans* AnVelB: ABQ17967; *Aspergillus niger* AniVelB: XP_001389053; *Aspergillus oryzae* AoVelB: EIT80146; *Coccidioides posadasii* CpVelB: EFW13315; *Paracoccidioides brasiliensis* PbVelB: EEH43217; *Penicillium roqueforti* PrVelB: CDM27432; *Trichophyton equinjum* TeVelB: EGE01484; *Talaromyces stipitatus* TsVelB: XP_002482693; *Uncinocarpus reesii* UrVelB: XP_002585361. **Sordariomycetes**: *Fusarium fujikuroi* FfVelB: CBK25977; *Fusarium oxysporum* FoVelB: ENH71845; *Verticillium dahlia* VdVelB: EGY18381. **Dothideomycetes**: *Curvularia lunata* ClVelB: KY435512; *Dothistroma septosporum* DsVelB: EME39661; *Pyrenophora tritici-repentis* PtVelB: XP_003303963; *Setosphaeria turcica* StVelB: XP_008031234. **Basidiomycetes**: *Schizophyllum commune* ScVelB: XP_003038135; *Laccaria bicolor* LbVelB: XP_001876565; *Moniliophthora roreri* MrVelB: XP_007845612.

**How to cite this article:** Gao, J.-X. *et al*. Involvement of a velvet protein ClVelB in the regulation of vegetative differentiation, oxidative stress response, secondary metabolism, and virulence in *Curvularia lunata. Sci. Rep.* **7**, 46054; doi: 10.1038/srep46054 (2017).

**Publisher's note:** Springer Nature remains neutral with regard to jurisdictional claims in published maps and institutional affiliations.

## Supplementary Material

Supplementary Table S1

## Figures and Tables

**Table 1:** Growth rate of *C. lunata* mutants compared with that of WT.

Strain	Light	Cycle	Dark
WT	6.07 ± 0.07	6.31 ± 0.16	6.41 ± 0.28
ΔClVelB	5.56 ± 0.05b	5.51 ± 0.17b	5.22 ± 0.10c
ClVelB-C	5.98 ± 0.05a	6.10 ± 0.02a	5.60 ± 0.07b

The diameter of hyphal radii at day 7 after incubation on CM plates at 28 °C. The data in all columns are the means of three independent experiments with standard deviation. The statistical analysis was performed using the SAS statistical package. Statistically significant analysis of variance (ANOVA) was further analyzed using least significant difference (LSD) tests. Different letters in each data column indicate significant differences at *P* = 0.05.

## References

[b1] Liu T. . Clg2p interacts with Clf and ClUrase to regulate appressorium formation, pathogenicity and conidial morphology in *Curvularia lunata*. Sci. Rep. 6, 24047 (2016). 27041392 10.1038/srep24047 PMC4819193

[b2] Liu T., Liu L. X., Jiang X., Huang X. L. & Chen J. A new furanoid toxin produced by *Curvularia lunata*, the causal agent of maize *Curvularia* leaf spot. Can. J. Plant Pathol. 31, 22–27 (2009).

[b3] Gao J. X., Liu T. & Chen J. Insertional mutagenesis and cloning of the gene required for the biosynthesis of the non-host specific toxin in *Cochliobolus lunatus* that causes maize leaf spot. Phytopathology 104(4), 332–339 (2014). 24134718 10.1094/PHYTO-07-13-0190-R

[b4] Gao S. G. . Understanding resistant germplasm-induced virulence variation through analysis of proteomics and suppression subtractive hybridization in a maize pathogen *Curvularia lunata*. Proteomics 12, 1–12 (2012). 23044763 10.1002/pmic.201200105

[b5] Merhej J., Richard-Forget F. & Barreau C. Regulation of trichothecene biosynthesis in *Fusarium*: recent advances and new insights. Appl. Microbiol. Biotechnol. 91, 519–528 (2011). 21691790 10.1007/s00253-011-3397-x

[b6] Bayram O. . VelB/VeA/LaeA complex coordinates light signal with fungal development and secondary metabolism. Science 320, 1504–1506 (2008). 18556559 10.1126/science.1155888

[b7] Wiemann P. . FfVel1 and FfLae1, components of a velvet-like complex in *Fusarium fujikuroi*, affect differentiation, secondary metabolism and virulence. Mol. Microbiol. 77, 972–994 (2010). 20572938 10.1111/j.1365-2958.2010.07263.x PMC2989987

[b8] Yang Q., Chen Y. & Ma Z. Involvement of BcVeA and BcVelB in regulating conidiation, pigmentation and virulence in *Botrytis cinerea*. Fungal Genet. Biol. 50, 63–71 (2013). 23147398 10.1016/j.fgb.2012.10.003

[b9] Eliahu N., Igbaria A., Rose M. S., Horwitz B. A. & Lev S. Melanin biosynthesis in the maize pathogen *Cochliobolus heterostrophus* depends on two mitogen-activated protein kinases, *Chk1* and *Mps1*, and the transcription factor Cmr1. Eukaryot Cell 6, 421–429 (2007). 17237364 10.1128/EC.00264-06 PMC1828933

[b10] Gao J. X., Jing J. & Chen J. Elementary coordinated expression research on genes related to the synthesis of pathogenesis-related melanin and toxin in *Cochliobolus lunatus*. J. SJTU. Agr. Sci. 33 (4), 53–58 (2015).

[b11] Wojda I., Alonso-Monge R., Bebelman J. P., Mager W. H. & Siderius M. Response to high osmotic conditions and elevated temperature in *Saccharomyces cerevisiae* is controlled by intracellular glycerol and involves coordinate activity of MAP kinase pathways. Microbiol.-SGM 149, 1193–1204 (2003). 10.1099/mic.0.26110-0 12724381

[b12] Roncero C. & Duran A. Effect of calcofluor white and Congo red on fungal cell wall morphogenesis – *in vivo* activation of chitin polymerization. J. Bacteriol. 163, 1180–1185 (1985). 3897187 10.1128/jb.163.3.1180-1185.1985 PMC219256

[b13] Wosten H. A., Richter M. & Willey J. M. Structural proteins involved in emergence of microbial aerial hyphae. Fungal Genet. Biol. 27, 153–160 (1999). 10441441 10.1006/fgbi.1999.1130

[b14] Jiang J. H., Yun Y. Z., Liu Y. & Ma Z. H. *FgVELB* is associated with vegetative differentiation, secondary metabolism and virulence in *Fusarium graminearum*. Fungal Genet. Biol. 49, 653–662 (2012). 22713714 10.1016/j.fgb.2012.06.005

[b15] Calvo A. M. The VeA regulatory system and its role in morphological and chemical development in fungi. Fungal Genet. Biol. 45, 1053–1061 (2008). 18457967 10.1016/j.fgb.2008.03.014

[b16] Bayram O. S. . LaeA control of velvet family regulatory proteins for light-dependent development and fungal cell-type specificity. PLos Genetics 6, e1001226 (2010). 21152013 10.1371/journal.pgen.1001226 PMC2996326

[b17] Park H. S. . Velvet-mediated repression of β-glucan synthesis in *Aspergillus nidulans* spores. Sci. Rep. 5, 10199 (2015). 25960370 10.1038/srep10199 PMC4426670

[b18] Kim H. J., Han J. H., Kim K. S. & Lee Y. H. Comparative functional analysis of the velvet gene family reveals unique roles in fungal development and pathogenicity in *Magnaporthe oryzae*. Fungal Genet. Biol. 66, 33–43 (2014). 24632440 10.1016/j.fgb.2014.02.011

[b19] Gao J. X. . Identification of proteins associated with the production of melanin and with pathogenicity in maize pathogen *Curvularia lunata*. Australas. Plant Path. 44(6), 599–603 (2015).

[b20] Wu D. L., Oide S., Zhang N., Choi M. Y. & Turgeon B. G. ChLae1 and ChVel1 Regulate T-toxin production, virulence, oxidative stress response, and development of the maize pathogen *Cochliobolus heterostrophus*. PLos Pathog. 8(2), e1002542 (2012). 22383877 10.1371/journal.ppat.1002542 PMC3285592

[b21] Gao J. X., Jing J., Liu T. & Chen J. Identification of Clt-1-regulated proteins associated with the production of non-host-specific toxin and pathogenicity in *Cochliobolus lunatus*. J. Phytopathol. 163(1), 33–41 (2015).

[b22] Sarikaya-Bayram Ö., Palmer J. M., Keller N., Braus G. H. & Bayram Ö. One Juliet and four Romeos: VeA and its methyltransferases. Front. Microbiol. 6, 00001 (2015). 10.3389/fmicb.2015.00001 PMC4299510 25653648

[b23] Lee M. K. . Negative regulation and developmental competence in *Aspergillus*. Sci. Rep. 6, 28874 (2016). 27364479 10.1038/srep28874 PMC4929475

[b24] Gao S. G. . Genome sequence and virulence variation-related transcriptome profiles of *Curvularia lunata*, an important maize pathogenic fungus. BMC Genomics 15, 627 (2014). 25056288 10.1186/1471-2164-15-627 PMC4124159

[b25] Gao S. G., Zhou F. H., Liu T., Li Y. Y. & Chen J. A MAP kinase gene, *Clk1*, is required for conidiation and pathogenicity in the phytopathogenic fungus *Curvularia lunata*. J. Basic Microbiol. 53(3), 214–223 (2012). 22733544 10.1002/jobm.201100518

[b26] Fan L. L. . Thc6 protein, isolated from *Trichoderma harzianum*, can induce maize defense response against *Curvularia lunata*. J. Basic Microbiol. 54, 1–10 (2014). 24771614 10.1002/jobm.201300814

[b27] Oide S. . Histidine kinase two component response regulator proteins regulate reproductive development, virulence, and stress responses of the fungal cereal pathogens *Cochliobolus heterostrophus* and *Gibberella zeae*. Eukaryot Cell 9, 1867–1880 (2010). 21037181 10.1128/EC.00150-10 PMC3008274

[b28] Wheeler M. H. & Greenblatt G. A. The inhibition of melanin biosynthetic reactions in *Pyricularia-oryzae* by compounds that prevent rice blast disease. Exp. Mycol. 12, 151–160 (1988).

[b29] Noh J. M. . Kojic acid-amino acid conjugates as tyrosinase inhibitors. Bioorg Med. Chem. Lett. 19, 5586–5589 (2009). 19700313 10.1016/j.bmcl.2009.08.041

[b30] Livak K. J. & Schmittgen T. D. Analysis of relative gene expression data using real-time quantitative PCR and the 2(-Delta Delta C(T)) Method. Methods 25, 402–408 (2001). 11846609 10.1006/meth.2001.1262

[b31] Fields S. & Song O. A novel genetic system to detect protein-protein interactions. Nature 340, 245–246 (1989). 2547163 10.1038/340245a0

