# Supplementary material for: Involvement of a velvet protein ClVelB in the regulation of vegetative differentiation, oxidative stress response, secondary metabolism, and virulence in Curvularia lunata
Source: Sci Rep. 2017 Apr 10;7:46054. doi: 10.1038/srep46054 (PMC5385503; doi:10.1038/srep46054)
Supplement: Supplementary Table S1 [file srep46054-s1.pdf]

# Involvement of a velvet protein ClVelB in the regulation of vegetative differentiation, oxidative stress response, secondary metabolism, and virulence in *Curvularia lunata*

**Jin-Xin Gao[1,2,3], Chuan-Jin Yu[1,2,3], Meng Wang[1,2,3], Jia-Nan Sun[1,2,3], Ya-Qian Li[1,2,3] & Jie Chen[1,2,3]**

| Primers | Sequence 5' to 3' | Description | PCR (kb) [a] | Purpose |
|---|---|---|---|---|
| VelB-FL-F | ATGGCCGCTAGCATGGCTCA | *clvelB* coding region, FP [b] | 1.011 | [d] ΔClVelB and ClVelB-C confirmation |
| VelB-FL-R | CTAGTCATCGGCGTCGTACT | *clvelB* coding region, RP [c] | | |
| VelBUp_F | <u>CCCAAGCTT</u>CACGGTTGAGGCAGACAT | flanking region upstream of *clvelB* FP | 0.940 | ΔClVelB |
| VelBUp_R | <u>ACGCGTCGAC</u>GGTCGTCGTCGTCGTCGT | flanking region upstream of *clvelB* RP | | |
| VelBDown_F | <u>CGGGGTACC</u>CCATTCCACACGCTCTCCAT | flanking region downstream of *clvelB* FP | 0.968 | ΔClVelB |
| VelBDown_R | <u>CCGGAATTC</u>GGTCGGCGGAAACATA | flanking region downstream of *clvelB* RP | | |
| hyg-F | CGACAGCGTCTCCGACCTGA | *hph* coding region, FP | 0.811 | ΔClVelB confirmation |
| hyg-R | CGCCCAAGCTGCATCATCGAA | *hph* coding region, RP | | |
| VelB-PVelB-F | <u>CCAAGCTT</u>CACATCCAATCTCTCAG | upstream of *clvelB* promoter, FP | 2.506 | ClVelB-C |
| VelB-PVelB-R | <u>GCTCTAGA</u>GTCATCGGCGTCGTACTCTG | *clvelB* coding region, RP | | |
| VelB-hbT_F | <u>GCTCTAGA</u>ACTTAACGTTACTGAAATC | TrpC terminator, FP | 0.481 | |
| VelB-hbT_R | <u>CGGAATTC</u>AACCCAGGGGCTGGTGACG | TrpC terminator, RP | | |
| G418_F | ACGCAGGTTCTCCGGCCGC | *G418* coding region, FP | 0.770 | ClVelB-C confirmation |
| G418_R | ACCTGAGCGAAATCAAGGACT | *G418* coding region, RP | | |
| GAPDH_2F | TCGTCGCCGTAAACGACCCC | *gapdh* coding region, FP | 0.207 | qRT-PCR |
| GAPDH_2R | CGCCCTTGAACTGGCCGTGT | *gapdh* coding region, RP | | |
| Brn1-1F | TGGCCAGCCAGTAGACATTG | *brn1* coding region, FP | 0.075 | |
| Brn1-1R | ACCTTTCCGTTGACCCACTC | *brn1* coding region, RP | | |
| Brn2-2F | AACAACGGCCGTATCATCCT | *brn2* coding region, FP | 0.078 | |
| Brn2-2R | AGCGTTGTAAAGAGCGTGGT | *brn2* coding region, RP | | |
| CMR1-1F | GTTTGGACTGACTCGCTGGT | *cmr1* coding region, FP | 0.118 | |
| CMR1-1R | TAGGATGATCGGCGGGAAGA | *cmr1* coding region, RP | | |
| SCD_1F | CGGTCGTTCCTGGACAAGATG | *scd* coding region, FP | 0.121 | |
| SCD_1R | GTGTGCCTCCGATAAAGTGCTG | *scd* coding region, RP | | |
| CAT3-1F | GACGATTGGTGTCAATGCCG | *cat3* coding region, FP | 0.140 | |
| CAT3-1R | GGACAAGAAGGTCGGCTTGA | *cat3* coding region, RP | | |
| Y2H-VelB-F | <u>CCGGAATTC</u>ATGGCCGCTAGCATGGCT | *clvelB* coding region, FP | 1.011 | Y2H |
| Y2H-VelB-R | <u>CGCGGATCC</u>CTAGTCATCGGCGTCGTA | *clvelB* coding region, RP | | |

| | | | | |
|---|---|---|---|---|
| Y2H-VeA-F | CGGAATTCATGTCCAACATCGTGGT | *clveA* coding region, FP | 1.797 | |
| Y2H-VeA-R | GCGGATCCTTAGACGCCGACTTCGGGTA | *clveA* coding region, RP | | |
| Y2H-VosA-F | CGGAATTCATGCCATATCACCAACCA | *clvosA* coding region, FP | 0.909 | |
| Y2H-VosA-R | GCGGATCCTCACATGTACTGATCGAA | *clvosA* coding region, RP | | |
| attL-VelBF | GGGGACAAGTTTGTACAAAAAAGCAGGC<br>TTCATGGCCGCTAGCATGGCT | *clvelB* coding region, FP | 1.008 | BiFC |
| attL-VelBR | GGGGACCACTTTGTACAAGAAAGCTGGG<br>TCGTCATCGGCGTCGTACTCT | *clvelB* coding region, RP | | |
| attL-VeAF | GGGGACAAGTTTGTACAAAAAAGCAGGC<br>TTCATGTCCAACATCGTGGTCA | *clveA* coding region, FP | 1.794 | |
| attL-VeAR | GGGGACCACTTTGTACAAGAAAGCTGGG<br>TCGACGCCGACTTCGGGTACGT | *clveA* coding region, RP | | |
| attL-VosAF | GGGGACAAGTTTGTACAAAAAAGCAGGC<br>TTCATGCCATATCACCAACCACA | *clvosA* coding region, FP | 0.906 | |
| attL-VosAR | GGGGACCACTTTGTACAAGAAAGCTGGG<br>TCCATGTACTGATCGAACATGT | *clvosA* coding region, RP | | |

**Table S1. Primer used for this study.** [a] PCR (kb) = PCR product length in kb; [b] FP = forward primer; [c] RP = reverse primer; [d]$\Delta$ = gene deletion. *The underlined regions identify the added restriction sites.
